# Tomato RNA-seq Data Mining Reveals the Taxonomic and Functional Diversity of Root-Associated Microbiota

**DOI:** 10.3390/microorganisms8010038

**Published:** 2019-12-24

**Authors:** Matteo Chialva, Stefano Ghignone, Mara Novero, Wael N. Hozzein, Luisa Lanfranco, Paola Bonfante

**Affiliations:** 1 Department of Life Sciences and Systems Biology, University of Torino, Viale P.A. Mattioli 25, I-10125 Torino, Italy; mara.novero@unito.it (M.N.); luisa.lanfranco@unito.it (L.L.); paola.bonfante@unito.it (P.B.); 2 Institute for Sustainable Plant Protection, National Research Council of Italy (CNR), Viale P.A. Mattioli 25, I-10125 Torino, Italy; stefano.ghignone@ipsp.cnr.it; 3 Bioproducts Research Chair, Zoology Department, College of Science, King Saud University, 11451 Riyadh, Saudi Arabia; whozzein@ksu.edu.sa; 4 Botany and Microbiology Department, Faculty of Science, Beni-Suef University, Beni-Suef 62511, Egypt

**Keywords:** fungi, holobiont, meta-transcriptome, microbiota, RNA-seq, tomato

## Abstract

Next-generation approaches have enabled researchers to deeply study the plant microbiota and to reveal how microbiota associated with plant roots has key effects on plant nutrition, disease resistance, and plant development. Although early “omics” experiments focused mainly on the species composition of microbial communities, new “meta-omics” approaches such as meta-transcriptomics provide hints about the functions of the microbes when interacting with their plant host. Here, we used an RNA-seq dataset previously generated for tomato (*Solanum lycopersicum*) plants growing on different native soils to test the hypothesis that host-targeted transcriptomics can detect the taxonomic and functional diversity of root microbiota. Even though the sequencing throughput for the microbial populations was limited, we were able to reconstruct the microbial communities and obtain an overview of their functional diversity. Comparisons of the host transcriptome and the meta-transcriptome suggested that the composition and the metabolic activities of the microbiota shape plant responses at the molecular level. Despite the limitations, mining available next-generation sequencing datasets can provide unexpected results and potential benefits for microbiota research.

## 1. Introduction

”Omics” approaches have profoundly changed our views on environmental and host-associated microbiotas. Metagenomics has allowed researchers to explore the diversity and composition of fungal and bacterial communities under diverse environmental conditions [1], and meta-transcriptomics identifies the most relevant metabolic pathways operating in the microbiota. Such approaches have been applied to the human microbiota, linking microbial profiles with the emergence of specific diseases [2], and to plant microbiota, describing the metabolic processes of the microbes associated with different species of plants [3,4]. Moreover, because of the large amount of information generated by next-generation sequencing (NGS), plant RNA-seq datasets that were originally generated to study the host transcriptome may be a novel resource for studying the plant-associated microbiota [5].

Some studies have focused on host–microbe pairs under controlled laboratory conditions, in so-called “dual RNA-seq” approaches and applied to plants such as cassava (*Manihot esculenta*) colonized by different arbuscular mycorrhizal (AM) fungal isolates [6] and Norway spruce (*Picea abies*) infected with *Heterobasidion annosum* [7]. Plant RNA-seq datasets have also been used to study the plant-associated microbiota, for example in the perennial grass *Holcus lanatus* growing in different soils [8]. These approaches are often limited by sequencing depth or read length, requiring assembly and annotation of the reads [5,9], and may have potential drawbacks such as the assembly of chimeric transcripts [10]. They demonstrate, however, how unbiased RNA-sequencing may reveal not only the biochemical functions being performed by a complex microbial community, but also the species within the community that are metabolically active.

Here, we used an RNA-seq dataset previously generated for tomato plants (*Solanum lycopersicum*) growing on different native soils [11,12] to test the hypothesis that host-targeted transcriptomics can detect the taxonomic and functional diversity of the root microbiota. We also examined potential correlations of microbiome diversity and activity with specific plant gene expression profiles. The previous research, which integrated transcriptomics and proteomics, was focused on the plant side—it revealed that two tomato cultivars, growing in native soils with different biological properties, responded to natural soil microbiotas by increasing their innate immunity and activating basal defense responses. The transcriptomic response of the plants depended more on soil type, and likely on the microbiota present, than on plant genotype. Parallel investigations of the cultivable fungi and of the tomato-associated mycobiota using internal transcribed spacer (ITS)-metabarcoding revealed consistent differences in the fungal community assemblage depending on the soil and plant genotype [13,14]. Here, we focus our attention on the microbial side, covering both fungal and bacterial communities.

To verify the feasibility of our approach, we developed a bioinformatic pipeline that was able to taxonomically and functionally annotate short RNA-seq reads. The pipeline takes advantage of read alignment, lowest common ancestor assignments, and functional annotation to reconstruct the diversity of active microbes associated with the roots of the two tomato cultivars grown on different native soils or on a control substrate. Our analysis succeeded in rendering bacterial and fungal diversity profiles, as well as identifying active functional categories. Applying the pipeline to our system we verified the hypothesis that microbial functions are also impacted by plant pathogen tolerance and soil suppressiveness. Finally, using genome-wide RNA-seq data mapped on tomato we further correlated the diversity and functional profiles to the host transcriptome.

## 2. Materials and Methods

### 2.1. Plant Material and Sequencing

The RNA-seq libraries analyzed in this study were obtained in a previous study [11]. The experimental setup consisted of two tomato (*Solanum lycopersicum*) genotypes, “Battito” (B) and “Cuore Di Bue” (C), which are resistant and susceptible, respectively, to *Fusarium oxysporum* f.sp. *lycopersici* (FOL) pathogen (races 1 and 2). Both genotypes were grown in a greenhouse pot experiment in two field-sampled soils, “Albenga” (AL) and “Rosta” (RO), which are suppressive and conducive, respectively, to FOL and in a disinfected peat-based substrate as a control (CONT). For each condition, three biological replicates were considered. A complete characterization of soil physicochemical features is available in Poli et al. [13]. Details of the sampling protocol, RNA extraction, and Illumina sequencing (50 bp single-end) procedures are described in Chialva et al. [11]. Raw RNA-seq reads are available in the NCBI Short Read Archive repository [15] under accession no. SRP126554. The same dataset was used to analyze the tomato transcriptome and to reconstruct the microbial diversity associated with plant roots as described below.

### 2.2. Plant Transcriptome Analysis

Raw RNA-seq libraries were analyzed according to the bioinformatic pipeline described in Chialva et al. [12], using the Heinz tomato genome (SL2.4) as the reference [16] and normalizing expression values with the DESeq2 package [17] in R [18].

### 2.3. Meta-Transcriptome Filtering and Annotation

Reads originating from microbial populations were filtered and annotated using a custom bioinformatic pipeline. Because the short read length could lead to the assembly of chimeric sequences, we chose to annotate reads rather than generating an assembly.

Raw adapter-filtered reads were first trimmed using TRIMMOMATIC v0.35 [19] setting Q >28 (with a 10-base sliding window) and length >45 bp. Host reads were then removed by mapping trimmed libraries on the tomato reference genome (SL.2.50) [16] using STAR c2.2.5a [20] in “end-to-end” mode, allowing a maximum number of 1000 multi-reads and setting intron length limits to 40 and 23,000 bp. To remove residual host reads, accounting for transcript variants and possible contaminants, we mapped reads on a NCBI NT subset containing all tomato and human sequences using Bowtie2 v2.2.9 [21] in “end-to-end” mode with “-D 25 -R 4 -N 1 -L 15 -i S,1,0.50” parameters. The rRNA sequences were then removed using Sortmerna v2.1 [22], using “-L 14 -passes 14,7,1 -e 1” parameters and the built-in databases plus a comprehensive subset of tomato rRNAs from the NCBI NT database [23] as the reference.

Filtered reads were then annotated on the whole NCBI NT database (release 13 July 2016) using the lowest common ancestor method (LCA) implemented by Taxoner64 v1.7 [24] according to the complete NCBI taxonomy (release 13 July 2016). Taxoner64 was run using the previously used Bowtie2 parameters (see above) and assigning reads to the nearest neighbor taxon ID at 97% sequence similarity.

Output files, containing reads annotated by taxid and GI (now accession number), were further processed in R [18] with custom scripts to obtain read counts per taxa. Taxonomic annotation for each taxon was retrieved using the CHNOSZ library [25], and data were further filtered to remove residual contaminants (Viridiplantae and Metazoa) as well as low-abundance taxa (<5 raw reads in at least 3 libraries).

As a further step, we functionally annotated the filtered microbial reads from the previous analysis by mapping to the eggNOG database v4.5 [26]. To overcome the short read length (50 bp), which did not allow functional assignment using conventional tools (such as MG-RAST or BLASTX), we generated a set of “pseudo-reads” extending reads 50 bp upstream and 50 bp downstream on their mapping reference in NCBI, thus generating longer 150 bp fragments. Pseudo-reads were generated taking into account reference sequence start and end coordinates and extracting selected ranges (150 bp) using the “getfasta” function in Bedtools v2.26.0-19 [27]. Pseudo-reads from different samples were then pooled and mapped on eggNOG using DIAMOND-BLASTX v0.8.24 [28] with -k1 -e0.001 -b15 -c1 parameters and removing redundant high-scoring segment pairs (HSPs).

### 2.4. Analysis of Arbuscular Mycorrhizal Colonization

Mycorrhizal colonization was assessed on a representative portion of the same root materials used to generate RNA-seq libraries. Immediately after sampling, roots were washed thoroughly under flowing tap water, stained for at least 12 h in methyl blue (0.1% *w*/*v* in lactic acid), and then bleached in lactic acid (3 washes, 1 h each). Root samples were then cut into 1 cm segments, mounted on glass slides, and observed by light microscopy. Mycorrhizal colonization parameters were visually estimated according to Trouvelot et al. [29]. For each condition, 9 different plants were analyzed, with 60 root segments examined for each plant.

### 2.5. Statistical Analysis

Statistical tests were performed in the R statistical programming environment [18]. Normalized read counts were grouped at the family level for bacteria and fungi. Permutational analysis of variance (PERMANOVA) of bacterial and fungal communities were computed using the “vegan” package v2.5-6 in R [30].

Differential abundance analysis was performed using the DESeq2 package v1.12.14 [17] to increase sensitivity relative to traditional rarefaction techniques [31]. The DESeq2 function was run using the independent filtering option and setting fitType = “parametric” and betaPrior = T. Taxa were considered as differentially abundant between conditions at an adjusted *p*-value < 0.05.

Statistical and differential Clusters of Orthologous Groups (COG) abundance analyses were obtained as previously described for taxa. Non-metric multidimensional scaling (NMDS) ordination analysis on functional categories was performed using “vegan” R package v2.5-6 [31]. Additionally, COG categories were mapped with KO KEGG identifiers using the “ko2cog.xl” mapping file at http://www.genome.jp/kegg/files/ko2cog.xl. Enriched KEGG pathways among KO IDs were then inferred using the clusterProfiler v3.14.0 package in R [32] at *p* < 0.1.

Data normality and homoscedasticity were tested using Shapiro–Wilk [33] and Levene’s test [34] in the “stats” v3.6.1 and “car” v3.0-3 packages [34], respectively (*p* < 0.05). According to data distributions, ANOVA was adopted for normal homoscedastic data and the Kruskal–Wallis test adopted for non-normal homoscedastic data [35] from the custom R package “stats” v3.6.1 at *p* < 0.05. Pairwise comparisons between treatments were performed when needed, using the appropriate post hoc tests. Tukey’s test [36] in the package “agricolae” v1.3-1 [37] was adopted for ANOVA, and Dunn’s test [38] in package “FSA” v0.8.25 [39] for Kruskall–Wallis, both at *p* < 0.05.

Variance partitioning analyses were used to explain plant transcriptome variance, using as explanatory variables meta-transcriptome diversity (fungi and bacteria) and functional diversity, as well as genotype and soil factors. DESeq2-normalized counts for microbial taxa and COG categories were used as meta-transcriptome descriptors. The analyses were performed using the “varpar” function in the “vegan” package [31]. Since collinearity was detected within datasets, a forward-selection procedure [40] was applied using the “forward.sel” function in the “adespatial” package v0.3-7 [41] with alpha = 0.05 and nperm = 999. Testable partitions were tested for significance using permutational ANOVA (999 permutation) on the RDA model (*p* < 0.05).

All analyses were performed on a HP Proliant server (64 cores, 128 GB RAM) running Ubuntu server 14.04. Custom R functions used to perform analyses are available at https://github.com/mchialva.

## 3. Results and Discussion

### 3.1. Reconstructing the Root-Associated Meta-Transcriptome from Host-Targeted RNA-seq Libraries

The RNA-seq dataset analyzed here was obtained from roots of two tomato genotypes grown in two native soils and one inert, control substrate in pots [11]. By observing the RNA-seq mapping rate onto the tomato reference genome, we noticed that a variable proportion of reads, ranging from 4% to 25%, were not aligning to the host genome. To uncover the diversity of active species in the microbial communities associated with tomato roots and their functional activities, we implemented a custom bioinformatic pipeline to filter reads generated by microbial transcripts (meta-transcriptome). The pipeline was composed of several steps, including reads trimming and filtering procedures, host reads and rRNA removal, and meta-transcriptome annotation (Figure S1). Taxonomic affiliations and functional annotations were obtained using the full NCBI Nucleotide database [23] and the eggNOG database [26], respectively.

**Figure 1:**
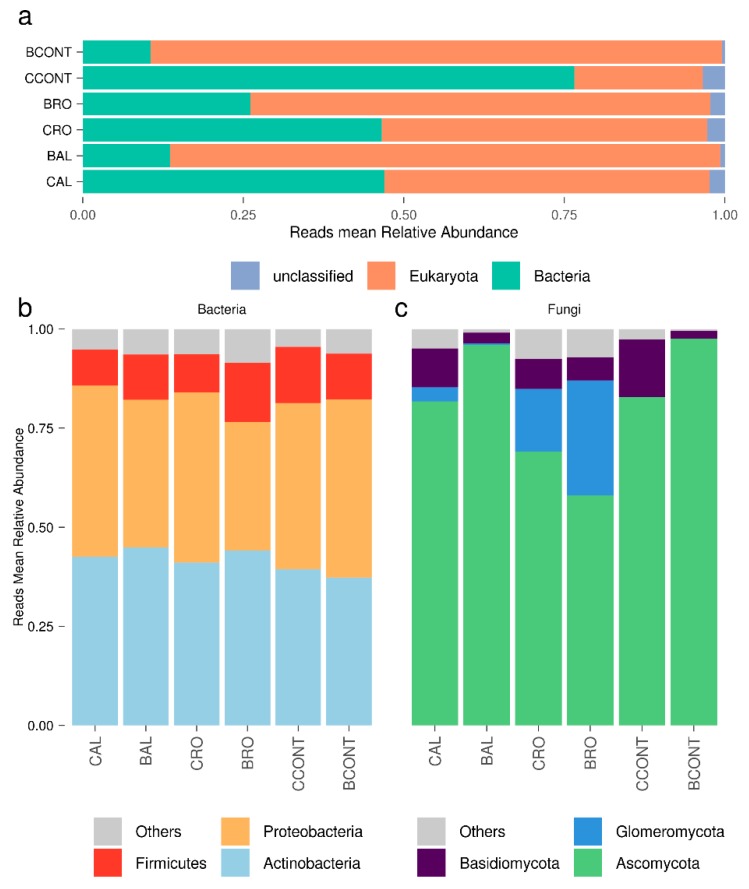
Active taxonomic diversity of root-associated microbiota of tomato plants grown in native and control soils. (**a**) Relative abundance of reads in each condition by superkingdom. (**b**,**c**) Bar plots of the three most represented phyla for bacteria (**b**) and fungi (**c**) among the different conditions. The first letter of the sample name indicates genotype (“Cuore di Bue” or “Battito”), while the following part refers to the soil/substrate type (RO, “Rosta” conducive soil; AL, “Albenga” suppressive soil; control (CONT), neutral peat-moss substrate).

Our pipeline detected from 93,736 to 581,439 microbial reads, depending on the sample, within the filtered sequences (Table 1). The vast majority of sequences (81% to 96%, depending on the library) were unique, displaying low redundancy. The mapping rate on the NCBI database ranged between 8.59% and 42.85% (Table 1), representing a variable proportion of bacteria and eukaryotes (Figure 1a).

Even though the Illumina libraries were enriched in poly-A transcripts, because they were initially targeted to the host plant, the analysis was successful in detecting a significant number of bacterial reads. The bacterial read counts were similar to those corresponding to fungi, possibly due to the presence of poly-A stretches in the bacterial transcripts. As frequently reported in similar studies, only a small proportion of the non-host reads could be successfully assigned [42,43], and in our case, this was probably due to the short read length (50 bp SE). Since the sampling protocol used to generate the RNA-seq dataset included repeated dH_2_O washes to remove most of the rhizospheric microbes [11], we hypothesized that the microbial meta-transcriptome reads could be attributed to both the rhizoplane and the endosphere root compartments. For this reason, we refer to these sequences as belonging to root-associated communities throughout. Indeed, all of the fungal and bacterial families we detected (see below) are well-known to be tightly associated with plant roots [44,45,46,47] and belong to diverse ecological guilds, from symbionts to saprotrophs.

### 3.2. Tomato Root-Associated Active Microbiota Diversity Is Shaped by Both Soil Type and Host Genotype

Most of the bacterial reads were assigned to Actinobacteria and Proteobacteria, while the fungal reads were dominated by Ascomycota (Figure 1b,c). At the phylum level (Figure 1b,c), a similar overall bacterial community composition emerged, with major variation across conditions observed only for fungi.

Indeed, the relative abundance of Glomeromycotina (Glomeromycota according to NCBI taxonomy) seemed to be dependent on soil type; this taxon was detected in both native soils but not in the control substrate. 

As expected, the analysis showed that root-associated communities also were present in plants growing on the disinfected substrate, demonstrating quick re-colonization of a microbe-depleted environment in the non-sterile greenhouse conditions used for these experiments.

The taxa-count tables we obtained for fungi and bacteria were then used to investigate the effect of soil versus plant genotype on microbial diversity (at the phylum and family levels). The PERMANOVA showed that host genotype explained a large part of the variance in community composition for bacteria (20.10% of variance explained, *p* < 0.01) and fungi (28.83%, *p* < 0.001) at the family level (Table 2). By contrast, soil type explained 20.25% of the variance (*p* < 0.05) only for the fungal community. These results are in agreement with data reported by Poli et al. [13] showing that the fungal communities tightly associated with tomato roots appeared to be mainly shaped by plant genotype.

We performed a differential abundance analysis comparing the “Cuore di Bue and “Battito” cultivars (Table S1). 

**Figure 2:**
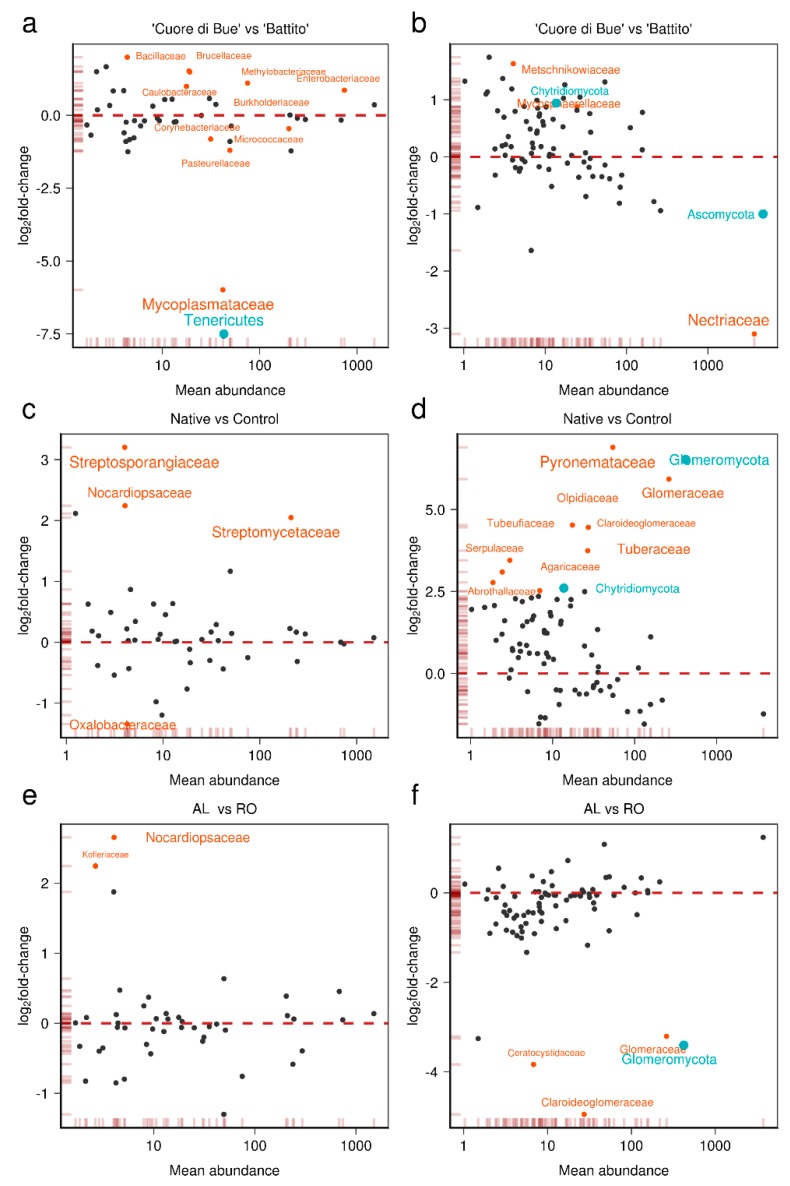
MA-plots of differential abundance analysis of bacterial and fungal families/phyla across genotype and soil types. (**a**,**b**) Enriched and depleted taxa of bacteria (**a**) and fungi (**b**) associated with “Cuore di Bue” versus “Battito” cultivars. (**c**,**d**) Enriched and depleted families of bacteria (**c**) and fungi (**d**) in native versus control soil. (**e**,**f**) Enriched and depleted families of bacteria (**e**) and fungi (**f**) in disease suppressive versus conducive soils. Differentially expressed (False Discovery Rate [FDR] < 0.05) families and phyla are depicted in pale red and light blue, respectively. Taxa name sizes are log_10_-proportional to the adjusted *p*-value (FDR). Rug density plots (pale red) along each axis indicate the density of abundances (*x*-axis) and log_2_fold-changes (*y*-axis); *x*-axis is log_10_-scaled.

Some Actinobacteria and Proteobacteria families that are commonly associated with the root endosphere and rhizosphere [48,49] were enriched or depleted in “Cuore di Bue” compared with “Battito” cultivars (Figure 2a). These families included Brucellaceae (log_2_FC = 1.48), Methylobacteriaceae (log_2_FC = 1.45), Burkholderiaceae (log_2_FC = 1.07), Pasteurellaceae (log_2_FC = −1.23), Alcaligenaceae (log_2_FC = −1.27), Corynebacteriaceae (log_2_FC = −0.84), and Micrococcaceae (log_2_FC = −0.48). Among the fungi, the susceptible cultivar “Cuore di Bue” hosted fewer Nectriaceae (Ascomycota), while other Ascomycota members, such as Mycospherellaceae, Metschnikowiaceae and Chytridiomycota were enriched (Figure 2b). Comparison of the two genotypes revealed a higher abundance in the resistant cultivar “Battito” of some fungal taxa such as Nectriaceae, which includes many *Fusarium* strains that can be plant pathogens or biocontrol agents. This result suggests that the resistant genotype readily recruits biocontrol Fusaria strains, which can putatively confer resistance against FOL as reported in the literature [50].

We also tested which taxa were enriched in native soils versus the neutral control. The results highlighted four bacterial families and 10 fungal families as differentially abundant in native soils (Table S1).

Among bacteria, Actinobacteria such as Streptosporangiaceae (log_2_FC = 3.19), Nocardiopsaceae (log_2_FC = 2.23), and Streptomycetaceae (log_2_FC = 2.04) emerged as predominant components of the native microbiota (Figure 2c). Among fungi (Figure 2d), Glomeraceae (log_2_FC = 5.97), Claroideoglomeraceae (log_2_FC = 4.46), Tuberaceae (log_2_FC = 3.77) and Pyronemataceae (log_2_FC = 6.89) were enriched in native soils relative to the control.

Compared to native soils, the control soil samples had a greater abundance of the bacterial family Oxalobacteraceae (log_2_FC = −1.34) (Figure 2c) but were not enriched for any fungal taxa (Figure 2c,d). Interestingly, Oxalobacteraceae, which is commonly found in soil and in association with the root endosphere, was previously described as one of the most persistent bacterial taxa associated with cucumber seeds [51]. If Oxalobacteraceae are similarly persistent in their association with tomato seeds, they might be the first to re-colonize the disinfected substrate (CONT), since no competition with any other microbes would exist.

The type of soil also influenced the root-associated microbiota. Specifically, we observed an enrichment of the bacterial families Nocardiopsaceae and Kofleriaceae in AL suppressive soil (Figure 2e). Interestingly, these two families were previously associated with soil disease suppressiveness because of their anti-fungal activity [52], confirming the disease-suppressive potential of AL soil. 

Among fungi (Figure 2f), arbuscular mycorrhizal (AM) fungi (Glomeraceae and Claroideoglomeraceae) as well as pathogenic fungi such as Ceratocystidaceae were less abundant in the AL disease-suppressive soil (False Discovery Rate [FDR] < 0.05).

**Figure 3:**
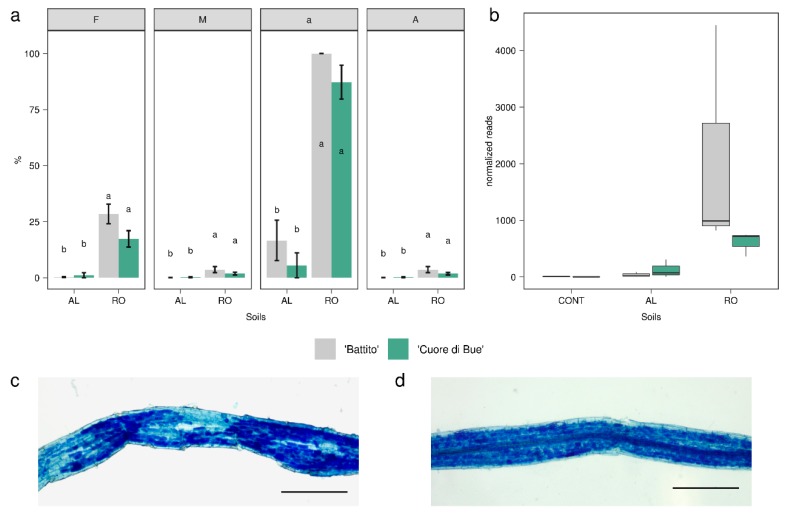
Mycorrhizal colonization of tomato roots growing in native soils and arbuscular mycorrhizal fungi (AMF) reads abundance. (**a**) Quantitative parameters of arbuscular mycorrhizal (AM) colonization of tomato roots (susceptible and resistant genotypes) growing in two different native soils: Mycorrhizal frequency (F), intensity of mycorrhization (M), presence of arbuscules within the mycorrhized segments (a), and the presence of arbuscules in the whole root apparatus (A) are plotted. Letters indicate significant differences among means according to the Kruskall–Wallis test (Dunn’s post hoc test, *p* < 0.05). (**b**) Normalized reads relative abundances assigned to phylum Glomeromycotina in the meta-transcriptome analysis. (**c**,**d**). Micrographs showing AMF colonization in tomato roots from AL (**c**) and RO (**d**) natural soils. Scale bars correspond to 30 μm in (**e**) and to 25 μm in (**f**).

*Funneliformis mosseae* was the most represented AM fungus. Interestingly, this species is cosmopolitan, able to colonize roots of a large number of different plants and is highly resistant to soil disturbances [53,54], since AM fungi are easily detectable by light microscopy after root staining, as we confirmed in the molecular results by examining root segments (Figure 3).

Quantitative analysis revealed a significant difference (*p* < 0.05) between RO and AL soils (Figure 3a), with a lower occurrence of fungal structures and arbuscules in roots of both tomato genotypes grown in AL suppressive soil. The decreased presence of AM fungi in the suppressive soil AL, along with a higher abundance of bacteria with well-known anti-fungal activities, raises novel questions about the fungal dynamics in suppressive soils [55].

### 3.3. Basal Microbial Metabolisms Are Detected in the Reconstructed Meta-Transcriptome

Reads assigned by the pipeline to bacterial and fungal taxa were pooled and functionally annotated to dissect microbial functional diversity associated with tomato roots under different soil and genotype conditions (Table S2). Reads were annotated using the eggNOG functional database [26] and differential expression analysis of Clusters of Orthologous Groups (COG) genes was performed. The mean mapping rate ranged between 13.9% and 59.10% of filtered reads (Table 1).

PERMANOVA analysis on COGs profiles (Table 2) showed that the largest part of the variation was linked to the soil type (*p* < 0.01, 24.63% explained variance), but also that the genotype has a relevant role (*p* < 0.05, 11.91% explained variance).

**Figure 4:**
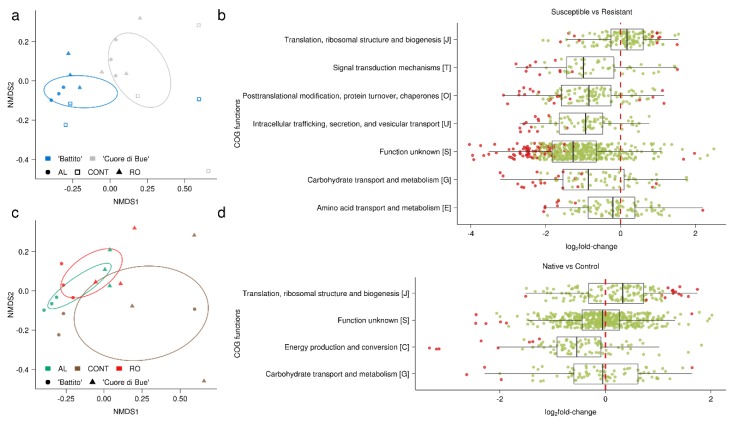
Meta-transcriptome functional diversity. (**a**,**c**) non-metric multidimensional scaling (NMDS) ordination plots on Bray–Curtis distances of expressed Clusters of Orthologous Groups (COG) functions clustered using 95% confidence ellipses by genotype (**a**) and substrate type (**c**); stress = 0.0776. AL, “Albenga” suppressive soil; RO, “Rosta” conducive soil; CONT, neutral control soil. (**b**,**d**) Boxplot of differentially expressed COG functions (FDR < 0.05) between genotypes (susceptible versus resistant, (**b**), and substrate type (native versus control soils, (**d**)). COGs are clustered by the more frequently represented functional categories, and points represent COG terms for each functional category; COGs that are significantly differentially expressed (FDR < 0.05) are indicated in red, COGs that are not in light green.

Distances between functional profiles were visualized through non-metric multidimensional scaling (NMDS) ordination analysis, clustering genotypes, and soil type (Figure 4a,c). The ordination was well-supported by PERMANOVA analysis, which detected an effect of genotype and soil. The plot revealed a different pattern between the two tomato genotypes, with a clear separation between them (Figure 4a). Similarly, soil types also clustered apart, with a large overlap between the two native soils and a clear separation of the control substrate (Figure 4c).

We next compared expression of COGs in susceptible (“Cuore di Bue”) versus resistant (“Battito”) cultivars (Table S2) and found 210 differentially expressed COGs, with 33 up-regulated and 177 down-regulated (FDR < 0.05) genes (Figure 4b). Interestingly, with few exceptions, most of the differentially expressed COGs were depleted in the susceptible cultivar “Cuore di Bue” (i.e., they were enriched in the resistant “Battito” cultivar). Differentially expressed genes mainly belonged to a few functional categories: “Function unknown (S)” (76 COGs); “Carbohydrate transport and metabolism (G)” (20 COGs); “Posttranslational modification, protein turnover, chaperones (O)” (20 COGs); “Intracellular trafficking, secretion, and vesicular transport (U)” (12 COGs); “Translation, ribosomal structure and biogenesis (J)” (12 COGs); “Signal transduction mechanisms (T)” (11 COGs); and “Amino acid transport and metabolism (E)” (10 COGs). Among the most interesting of the down-regulated gene categories we found in the “Cuore di Bue” cultivar were calcium calmodulin-dependent protein kinases (ENOG410XRMJ, ENOG410XNRX) and serine threonine protein kinases (COG0515, ENOG410XNPH), both of which are categories involved in signal transduction. 

When we analyzed native soils versus control, we found 49 differentially expressed COGs, with a similar number of up- and down-regulated categories (Figure 4d). Up-regulated COGs mostly belonged to the “Translation, ribosomal structure and biogenesis (J)” category (15/24 COGs). Down-regulated genes were assigned to a few other categories, such as “Energy production and conversion (C)”, “Function unknown (S)”, and “Carbohydrate transport and metabolism (G)”, with eight, six, and four COGs terms, respectively. Of the 15 upregulated COGs assigned to category “J”, five corresponded to ribosomal proteins (COG1717, COG1997, COG1631, COG1632, COG1358). Since ribosomal proteins have been proposed as markers of in situ growth rates in microbial communities [56], our data suggest that in native soils, as compared to the control substrate, the higher microbial abundance associated with plant roots is mirrored by a higher microbial metabolic activity. Despite the differences between the native soils and the control, we found no COGs that were differentially expressed between the suppressive and conducive soils. This supports the NMDS ordination analysis, which displayed high similarities between AL and RO COGs expression profiles.

The functional diversity analysis revealed only a few basal metabolic functions, indicating that the sequencing depth was insufficient to capture a full profile of expressed microbial genes. However, we were able to detect major differences in the root-associated microbiota between native versus artificial substrates—both in the level of metabolic activity and in the genes expressed—and between different plant genotypes.

### 3.4. Linking the Meta-Transcriptome with the Host Transcriptome

**Figure 5:**
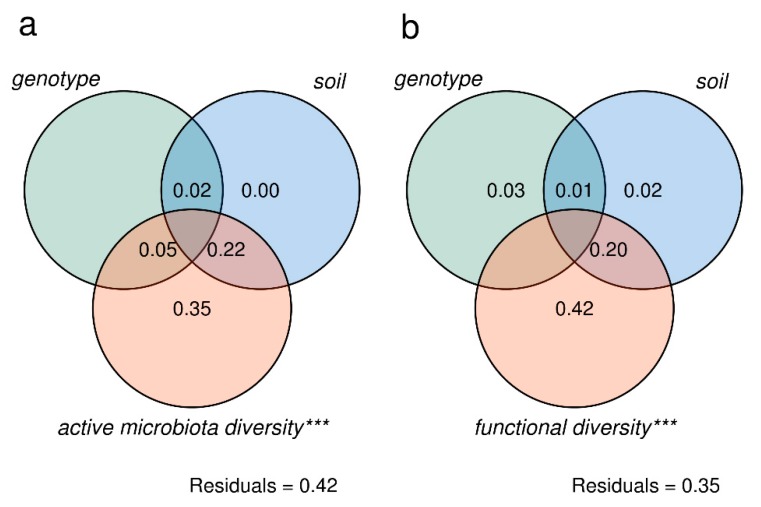
Variance partitioning analysis on meta-transcriptome and plant transcriptome profiling. (**a**) Tomato transcriptome variance explained by genotype, soil, and active microbiota diversity (bacteria and fungi). (**b**) Tomato transcriptome variance explained by genotype, soil, and mapped COG terms (functional diversity). Residuals, unexplained variance fractions (discrepancy between the data and an estimation model). Significant fractions are indicated by asterisks (ANOVA on RDA model, * *p* < 0.05; ** *p* < 0.01; *** *p* < 0.001).

Finally, we verified the feasibility of linking the taxonomic and functional diversity of the root-associated active microbiota, reconstructed from RNA-seq data, to the tomato transcriptome using variance-partitioning analysis. Meta-transcriptome features, namely LCA taxonomic assignments and functional diversity (expressed COG terms), were treated as explanatory variables of the variation in tomato expressed genes across conditions. We performed two independent analyses, first testing the influence of the taxonomic profile and then testing that of the functional profile (Figure 5). When considering the fungal and bacterial taxa associated with roots (Figure 5a), a large amount of plant transcriptome variance is explained by the active microbial diversity (35%, *p* < 0.001). By contrast, soil and genotype factors individually did not explain any of the transcriptome variance, although 2% of the explained variance was collinear to these two factors. Interestingly, 22% of the explained variance was collinear between soil type and microbial diversity, probably meaning that these two factors are interdependent in modulating tomato transcriptomic responses. Similarly, the genotype factor also shared 5% of collinearly explained variance with microbial diversity. The analysis was repeated considering active fungal and bacterial communities as independent factors. The bacterial community alone explained the greatest portion of plant transcriptome variance (18%) with high collinearity with fungal diversity (Figure S2). The active fungal community, however, did not explain a significant portion of the variance on its own, being entirely collinear with other factors (mainly soil type and bacterial community).

By using expressed COG functions within the meta-transcriptome as an explanatory variable, we were able to attribute 42% (*p* < 0.001) of the plant transcriptome variance to microbial functions (Figure 5b). We also detected collinearity between soil type and COG terms that amounted to 20%. However, unlike the results of the previous analysis, the variance explained exclusively by the soil factor dropped to 2% (*p* > 0.05).

These results highlight that the taxonomic composition and in particular the expression profile of root-associated microbial communities correlate well with the plant expression profile across soils and genotypes, explaining more variance than genotype and soil factors. However, unexpectedly, the cumulative contribution of soil type and COGs in explaining tomato gene expression was slightly higher, similar to what we described above when considering meta-transcriptome diversity. This suggests that there is a strong relationship between soil features and meta-transcriptome functioning/diversity.

## 4. Conclusions

Despite the low sequencing coverage of microbial transcripts, the deep mining of transcriptome datasets we previously generated from tomato plants grown in non-sterile environments [11] allowed the identification of a number of active microbes associated with roots. Our data demonstrate that this approach is feasible even with a high abundance of host sequences (>90%) and a short read length (50 bp), even though the number of microbial reads (excluding rRNAs) harvested was considerably lower than was obtained in other meta-transcriptome studies [57,58]. Notwithstanding these technical constraints, we were able to reconstruct the active microbiota and functional diversity as reported in other works [43]. The reliability of the mined information was validated only for Glomeromycotina, so the accuracy and precision of our whole-community assemblage reconstructions need to be further confirmed, such as by generating simulated RNA-seq libraries at different read lengths and coverage values.

When considering the active diversity and functional profiles of the tomato-associated microbes, we found that the main driver of both is the host genotype, largely confirming previous data obtained in tomato [13] as well as in other plant models, such as rice [59], *Arabidopsis* [49], and *Lotus* [60]. However, especially in terms of taxonomic diversity, we also found a major impact of soil type, which was more conspicuous when considering the fungal communities. In addition to many bacteria, we detected arbuscular mycorrhizal fungi in the roots of both genotypes, but these fungi were more abundant in tomatoes growing in conducive RO soil than in suppressive AL soil, as was also confirmed by morphological quantification. At the same time, pathogenic fungi were less represented in AL soil, where bacteria belonging to Nocardiopsaceae and Kofleriaceae, known for their potential anti-microbial activity [52], were dominant. Taken as a whole, the data provided detailed information on the diversity of microbial communities associated with the roots of two tomato genotypes and supported our previous findings [11,12]. Lastly, through variance partitioning analysis we showed a good correlation between meta-transcriptome (including both taxonomic and functional diversity of microbes associated with roots) and plant host transcriptome.

In conclusion, the use of host-targeted RNA-seq libraries to study the meta-transcriptome is a feasible approach. It allows the reconstruction of microbial taxonomical and functional diversity at a relatively low sequencing depth (>90% host sequences), maximizing the throughput of the RNA-seq approach in a cost-effective way.

## Figures and Tables

**Table 1:** Meta-transcriptome reads assignment statistics.

Sample	SRA ^1^ Run	SRA ^1^ Sample Accession	Raw	Filtered	Unique Filtered Reads (%)	Mapped on NCBI (%)	Bacteria	Eukaryotes	Mapped % (eggNOG)
BAL_R1	SRR6368019	SRS2751529	11,382,297	184,182	88.88	27.12	4952	16,074	33.41
BAL_R2	SRR6368032	SRS2751512	20,308,970	391,019	93.74	25.88	8662	34,437	34.28
BAL_R3	SRR6368031	SRS2751523	12,614,238	187,812	95.77	41.37	3141	54,951	59.10
BRO_R1	SRR6368030	SRS2751514	14,986,370	230,155	95.61	15.59	2909	7937	23.69
BRO_R2	SRR6368029	SRS2751516	15,367,923	207,560	95.43	17.80	2853	8265	18.92
BRO_R3	SRR6368036	SRS2751513	17,906,164	484,731	95.56	10.99	5185	13,803	29.70
BCONT_R1	SRR6368035	SRS2751519	12,893,929	93,736	85.68	19.14	5114	676	29.46
BCONT_R2	SRR6368034	SRS2751517	23,428,531	382,171	84.86	42.85	4751	98,337	44.00
BCONT_R3	SRR6368033	SRS2751518	13,161,547	117,290	91.33	34.91	2982	8861	23.11
CAL_R1	SRR6368022	SRS2751525	13,376,685	342,990	88.63	8.59	4691	7049	36.23
CAL_R2	SRR6368021	SRS2751526	12,343,880	317,136	81.39	13.11	9361	7628	34.98
CAL_R3	SRR6368024	SRS2751522	18,637,725	380,329	89.13	9.16	5928	6830	32.38
CRO_R1	SRR6368023	SRS2751524	13,977,334	208,810	90.82	10.80	4651	3801	31.17
CRO_R2	SRR6368026	SRS2751530	18,315,136	534,056	89.60	10.61	9998	13,518	34.78
CRO_R3	SRR6368025	SRS2751520	27,710,101	581,439	91.36	12.77	8804	8195	18.68
CCONT_R1	SRR6368028	SRS2751515	17,006,711	138,440	89.62	17.90	3071	820	13.90
CCONT_R2	SRR6368027	SRS2751521	29,267,531	246,552	92.28	22.81	11,392	2488	22.10
CCONT_R3	SRR6368020	SRS2751527	16,244,284	98,253	94.61	16.58	2804	1210	21.72

^1^ Short Read Archive respository at NCBI, https://www.ncbi.nlm.nih.gov/sra.

**Table 2:** PERMANOVA analysis of meta-transcriptome diversity and functioning in relation to host genotype, soil, and its interaction. Df = degrees of freedom; SS = sum of squares; MS = mean sum of squares; Pseudo-F = F value by permutation. Statistical significance is indicated in bold (*p* < 0.05); *p*-values are based on 999 permutations.

Source	Df	SS	MS	F	*R* ^2^	*p*	Explained Variance (%)
Bacteria (family)							
Genotype	1	0.10739	0.107388	4.5390	0.20102	0.0032	20.10
Soil	2	0.05908	0.029542	1.2487	0.11060	0.2511	11.06
Genotype × Soil	2	0.0838	0.041915	1.7716	0.15692	0.0762	15.69
Residual	12	0.28391	0.023659		0.53145		53.15
Total	17	0.53421			1		100
Fungi (family)							
Genotype	1	0.69477	0.69477	7.6321	0.28835	0.0004	28.83
Soil	2	0.48786	0.24393	2.6796	0.20247	0.0294	20.25
Genotype × Soil	2	0.13449	0.06724	0.7387	0.05581	0.6368	5.58
Residual	12	1.09239	0.09103		0.45337		45.34
Total	17	2.40951			1		100
COG genes							
Genotype	1	0.3782	0.3781	2.7336	0.11915	0.0253	11.92
Soil	2	0.7818	0.39089	2.8254	0.24630	0.0091	24.63
Genotype × Soil	2	0.3539	0.17696	1.2791	0.11151	0.2334	11.15
Residual	12	1.6601	0.13835		0.52304		52.30
Total	17	3.1740			1		100

## Supplementary Materials

The following are available online at https://www.mdpi.com/2076-2607/8/1/38/s1, Figure S1: Work-flow diagram of the bioinformatic pipeline used for meta-transcriptome analysis; Figure S2: Global variance partitioning analysis of the plant transcriptome profiling explained by meta-transcriptome fungal and bacterial diversity. Table S1: Raw meta-transcriptome read counts alongside their lowest common ancestor (LCA) annotation (taxa identifiers refer to NCBI taxonomy) and differential abundance contrasts performed using DESeq2 R package (FDR < 0.05) for bacterial and fungal families. Table S2: Meta-transcriptome reads functional assignment on eggNOG 4.5 database and differential expression analysis performed using DESeq2 R package (FDR < 0.05).
