# Supplementary material for: Tomato RNA-seq Data Mining Reveals the Taxonomic and Functional Diversity of Root-Associated Microbiota
Source: Microorganisms. 2019 Dec 24;8(1):38. doi: 10.3390/microorganisms8010038 (PMC7022885; doi:10.3390/microorganisms8010038)
Supplement: Supplementary file 1 [file microorganisms-08-00038-s001.zip › microorganisms-671253-supplementary.pdf]

Supplementary Materials

Supplementary Figures

Figure S1. Work-flow diagram of the bioinformatic pipeline used for meta-transcriptome analysis.

Figure S2. Global Variance Partitioning analysis of the plant transcriptome profiling explained by meta-transcriptome fungal and bacterial diversity.

Supplementary Tables

Table S1. Raw meta-transcriptome read counts alongside their LCA annotation (taxa identifiers refers to NCBI taxonomy) and differential abundance contrasts performed using DESeq2 R package (FDR<0.05) for bacterial and fungal families.

Table S2. Meta-transcriptome reads functional assignment on eggNOG 4.5 database and differential expression analysis performed using DESeq2 R package (FDR<0.05).

Supplementary Figures

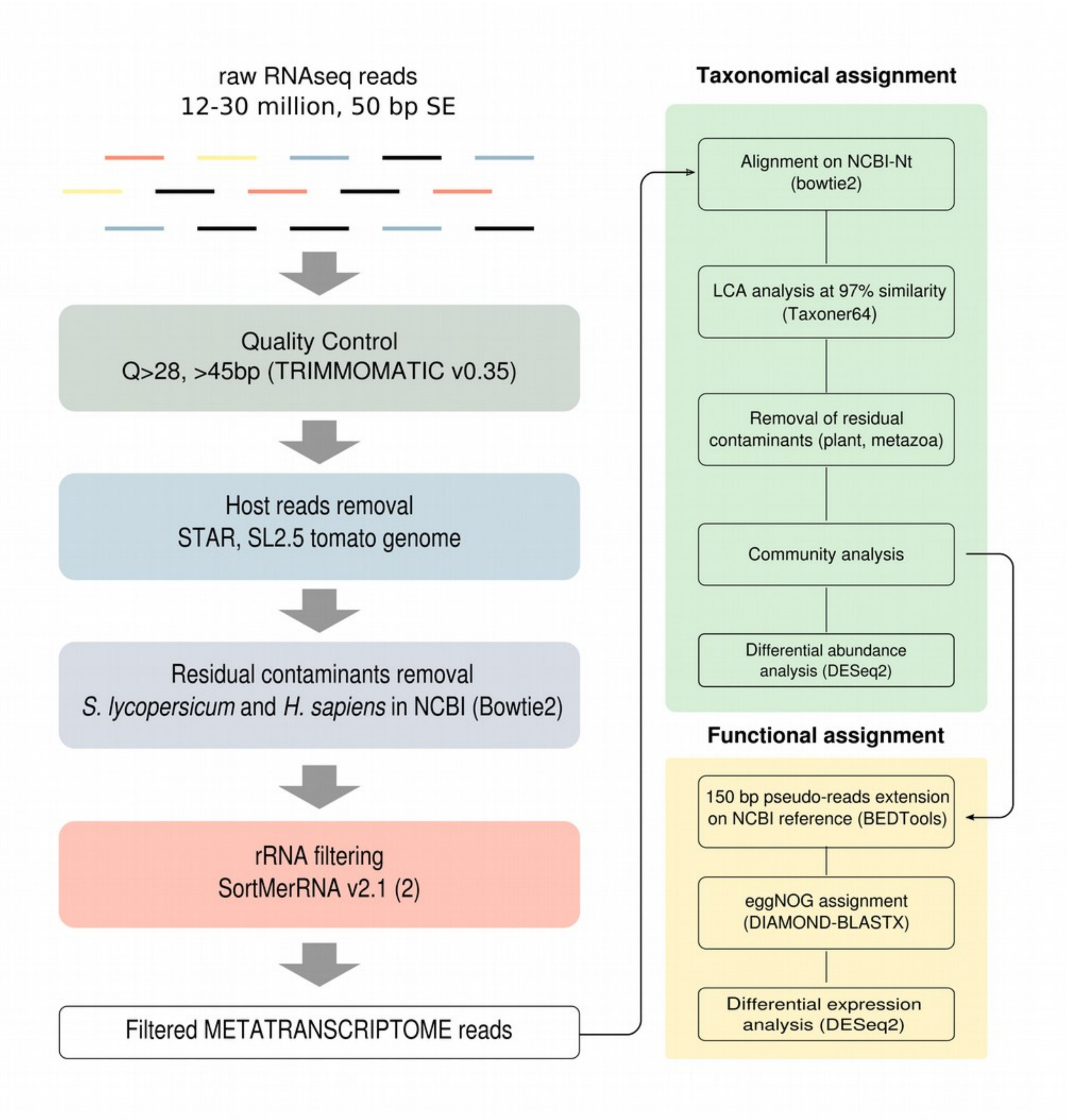

Figure S1. Work-flow diagram of the bioinformatic pipeline used for meta-transcriptome analysis.

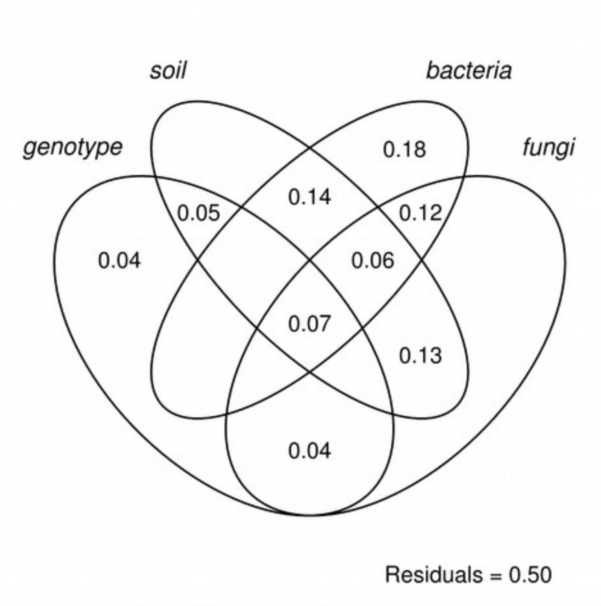

Figure S2. Global Variance Partitioning analysis of the plant transcriptome profiling explained by meta-transcriptome fungal and bacterial diversity. Forward-selected bacterial and fungal taxa matrices, soil type and genotype factors were used as explaining variables. None of the individual fraction resulted as significant in explaining plant transcriptome variance (ANOVA on RDA model, $P>0.05$).
